# Raman-microscopy investigation of vitrification-induced structural damages in mature bovine oocytes

**DOI:** 10.1371/journal.pone.0177677

**Published:** 2017-05-22

**Authors:** Giulia Rusciano, Carolina De Canditiis, Gianluigi Zito, Marcello Rubessa, Maria Serena Roca, Rosa Carotenuto, Antonio Sasso, Bianca Gasparrini

**Affiliations:** 1 Department of Physics “E. Pancini” - University of Naples Federico II, via Cintia, I-80126 Naples, Italy; 2 National Institute of Optics (INO)-National Research Council (CNR), Via Campi Flegrei 34, I-80078 Pozzuoli (NA), Italy; 3 Department of Veterinary Medicine and Animal Production, University of Naples Federico II, Via F. Delpino 1, I-80137 Naples, Italy; 4 Institute of Protein Biochemistry (IBP), National Research Council (CNR), Via Pietro Castellino, 111 - I-80131, Napoli, Italy; 5 Institute for Genomic Biology, University of Illinois, Urbana, IL, 61801, United States of America; 6 Experimental Pharmacology Unit, Istituto Nazionale Tumori “Fondazione G. Pascale” IRCCS, Napoli, Italy; 7 Department of Biology - University of Naples Federico II, via Cintia, I-80126 Naples, Italy; Seoul National University Bundang Hospital, REPUBLIC OF KOREA

## Abstract

Although oocyte cryopreservation has great potentials in the field of reproductive technologies, it still is an open challenge in the majority of domestic animals and little is known on the biochemical transformation induced by this process in the different cellular compartments. Raman micro-spectroscopy allows the non-invasive evaluation of the molecular composition of cells, based on the inelastic scattering of laser photons by vibrating molecules. The aim of this work was to assess the biochemical modifications of both the zona pellucida and cytoplasm of vitrified/warmed in vitro matured bovine oocytes at different post-warming times. By taking advantage of Principal Component Analysis, we were able to shed light on the biochemical transformation induced by the cryogenic treatment, also pointing out the specific role of cryoprotective agents (CPs). Our results suggest that vitrification induces a transformation of the protein secondary structure from the *α*-helices to the *β*-sheet form, while lipids tend to assume a more packed configuration in the zona pellucida. Both modifications result in a mechanical hardening of this cellular compartment, which could account for the reduced fertility rates of vitrified oocytes. Furthermore, biochemical modifications were observed at the cytoplasmic level in the protein secondary structure, with *α*-helices loss, suggesting cold protein denaturation. In addition, a decrease of lipid unsaturation was found in vitrified oocytes, suggesting oxidative damages. Interestingly, most modifications were not observed in oocytes exposed to CPs, suggesting that they do not severely affect the biochemical architecture of the oocyte. Nevertheless, in oocytes exposed to CPs decreased developmental competence and increased reactive oxygen species production were observed compared to the control. A more severe reduction of cleavage and blastocyst rates after in vitro fertilization was obtained from vitrified oocytes. Our experimental outcomes also suggest a certain degree of reversibility of the induced transformations, which renders vitrified oocytes more similar to untreated cells after 2 h warming.

## Introduction

Oocyte cryopreservation is fundamental for safeguarding genetic resources and for several potential applications in the field of reproductive technologies by increasing the availability of gametes for both research and commercial purposes. Although cryopreservation of mammalian oocytes has rapidly progressed during the past two decades [1], it still remains an open challenge in the majority of domestic animals. This is mainly due to the peculiar oocyte structure, exhibiting a low surface-to-volume ratio, which results in a high sensitivity to chilling and high susceptibility to intracellular ice formation. In addition, the permeability of the oocyte plasma membrane is low, delaying the movement of CPs and water [2]. There is a convincing evidence of the superiority of vitrification over slow freezing for oocyte cryopreservation in several species [3]. In order to achieve vitrification, cells are exposed to a high concentration of CPs and frozen with an ultra rapid cooling velocity, resulting in an ice crystal free, solid glass-like structure. Innovative vitrification methods employ minimum volumes of vitrification solution and direct contact with liquid nitrogen, in order to increase cooling and warming rates that are critical factors for a successful vitrification, allowing to reduce the CPs concentrations. Among these methods, Cryotop has been proven successful for cryopreserving oocytes of several species [4–6]. However, vitrification of oocytes is still challenging [7] and the efficiency of oocyte cryopreservation is still very low. In fact, despite the high survival rates after vitrification, low blastocyst rates from vitrified oocytes are widely reported.

Metaphase II (MII) oocytes remain the preferred stage for cryostorage due to better membrane stability during chilling procedures [8, 9]. It is well documented that the cryopreservation process induces several undesirable effects such as spindle disorganization, leading to disrupted chromosomes [10–12] and microfilaments [12]. Besides spindle damage, several factors might cause the reduced cleavage rates of vitrified oocytes, including their decreased ability to be penetrated by spermatozoa. The reason for the decreased rate of sperm penetration may be a structural change in the zona pellucida (ZP), resulting in zona hardening. Indeed, ultrastructural studies demonstrated that vitrification alters the ZP glycoprotein matrix and leads to ZP hardening by premature cortical granule exocytosis in bovine and human oocytes [13–15]. Changes in the membrane characteristics, caused by the cryopreservation-induced exocytosis of cortical granules, probably also play a role [16–18]. Different strategies have been developed to control these factors and increase oocyte viability and developmental competence, albeit with limited success [3, 8]. An important issue is the identification of the most appropriate time of insemination of vitrified-warmed oocytes before apoptosis occurs [19]. As some cryopreservation-induced damages are reversible, the optimal post-thawing interval before fertilization may play a critical role. In fact, several studies have shown that cryopreservation determines transient spindle disassembly, with the spindle recovered after a few hours of incubation [19]. The evaluation of oocyte molecular damage after vitrification/warming represents a crucial aspect of the overall evaluation of oocyte quality, providing information useful to plan corrective strategies. The analysis of oocyte should rely on methodologies ensuring sensitiveness, reproducibility and applicability, with an added value for rapid and non-invasive analyses. Recently, Raman Micro-spectroscopy (RMS) has been used to assess the changes caused by vitrification/warming of in vitro matured ovine oocytes [20]. RMS is a non-invasive technique that allows the evaluation of the molecular composition of cells, based on the inelastic scattering of laser photons by vibrating molecules [21]. Raman spectra provide information on the molecular bonds scattering the Raman beam, therefore constituting a sort of chemical fingerprint of the sample [22–24]. Aim of the present study was to investigate the structural modifications of ZP and cytoplasm of vitrified/warmed in vitro matured bovine oocytes at different post-warming times by RMS. For this purpose, Raman spectra from the ZP and cytoplasm of vitrified/warmed oocytes were compared to those corresponding to two control groups: (i) untreated oocytes and (ii) oocytes exposed to CPs but not to the cryogenic treatment. This choice allowed us to discriminate among the biochemical alterations due to the cryoinjury from those deriving from CPs toxicity. We also evaluated the time dependent biochemical changes after warming, in order to identify the most appropriate time for in vitro fertilization (IVF). Further objectives were to assess the oocyte developmental competence after IVF and to measure reactive oxygen species (ROS) in untreated, exposed to CPS and vitrified oocytes.

## Materials and methods

### Materials

Unless otherwise stated, all reagents were purchased from Sigma (Milan, Italy). The aspiration medium was Hepes-buffered TCM 199 supplemented with 2 mM sodium pyruvate, 1 mM L-glutamine, 10 *μ*L/mL amphotericin B (H199) supplemented with 2% adult BS and 95.6 SI/mL heparin. The medium was TCM 199 supplemented with 15% BS, 0.5 *μ*g/mL FSH, 5 *μ*g/mL LH, 0.8 mM L-glutamine and 50 *μ*g/mL gentamycin. The base medium for the vitrification and warming solutions was Hepes-buffered TCM199 with 20% fetal calf serum (FCS).

### Experimental design

In vitro matured oocytes were randomly divided into three experimental groups: vitrified/warmed oocytes (VITRI), oocytes only exposed to vitrification/warming solutions (CP) and control untreated oocytes (CTR). In order to assess time dependent biochemical changes following both vitrification/warming and exposure to CP, RMS analysis was carried out at different post-warming intervals. In particular, vitrified oocytes were warmed and incubated in the IVM medium for 0, 1, 2, 3 and 4 h. In order to assess the toxicity of CPs, in vitro matured oocytes were exposed to the vitrification solutions and directly processed through the warming solutions, bypassing vitrification, and then re-incubated in the IVM medium for 0, 1, 2, 3 and 4 h. Both VITRI and CP oocytes were fixed in 2% w/v paraformaldehyde for subsequent assessment at each warming interval, whereas the CTR oocytes were directly fixed at the end of IVM incubation. The size of each group analysed herein is reported in Table 1.

**Table 1:** Size of the 11 groups analysed in this work.

Group	Size	Group	Size	Group	Size
CTR	24	CP_0_	18	VITRI_0_	18
		CP_1_	14	VITRI_1_	15
		CP_2_	19	VITRI_2_	20
		CP_3_	16	VITRI_3_	18
		CP_4_	17	VITRI_4_	16

Furthermore, in order to evaluate the capability of the oocytes to undergo fertilization and embryo development, oocytes of the CTR group (n = 183) were fertilized at 22 h post-maturation, whereas the oocytes of the CP (n = 218) and VITRI (n = 218) groups were allowed 2 h of recovery from warming/exposure to CPs before insemination, as in current practice. This experiment was repeated 3 times. Finally, a representative number of oocytes of the three groups (n = 48/group, over 3 replicates) was used for ROS measurements at time 0 and at 2 h post-warming/exposure.

### Oocytes collection

Abattoir-derived oocytes were matured in vitro according to our standard procedure [25]. Briefly, bovine ovaries were recovered from a local abattoir ((Grottaminarda-Flumeri; Avellino, Italy) and transported to the laboratory in physiological saline at 30 to 35°C. Cumulus-oocyte complexes (COCs) were aspirated from follicles of 2 to 8 mm in diameter and only those with uniform cytoplasm and multilayered cumulus cells were selected, washed twice in the aspiration medium and once in the in in vitro maturation medium (IVM). Groups of 25 COCs were matured in 400 *μ*L of IVM, covered with mineral oil, in four well plates (NuncTM, Roskilde, Denmark), for 22 h at 39°C, and 5% CO_2_ in air.

### Oocyte vitrification and warming

After IVM, COCs were washed and mechanically stripped of their cumulus cells by vortex in H199 supplemented with 5% of BS. The denuded oocytes were vitrified by the Cryotop method, previously described [26]. The oocytes were incubated in 200 *μ*L drop of 10% (v/v) Ethylene Glycol (EG) + 10% (v/v) dimethyl sulfoxide (DMSO) for 3 min and then transferred into 50 *μ*l of 20% (v/v) Ethylene Glycol + 20% (v/v) DMSO + 0.5M Sucrose for 20-25 seconds. Groups of 4-6 oocytes were loaded with a glass capillary onto the top of the film strip of each Cryotop in a minimum volume. After loading, almost all the solution was removed (to an estimated final volume of < 0.1 *μ*L) and the Cryotop was quickly immersed in liquid nitrogen (within 25 seconds) for vitrification. For warming, a procedure of 4 steps with decreasing concentration of sucrose was used. The Cryotop strip was immersed directly into 1 ml of 1.25 M sucrose solution for 1 min, and the oocytes recovered were subsequently exposed to decreasing concentrations of sucrose (0.62 M, 0.42 M and 0.31 M) for 30 seconds each. After that, the oocytes were washed twice in 3 ml of H199 + 10% of FCS for 30-40 seconds and reallocated in the IVM medium for different time intervals according to the experiment.

### Raman confocal microscopy

Raman spectra were acquired by using a commercial Raman system (WiTec, alpha 300), described in details in ref. [27]. Briefly, it consists in a confocal microscope endowed with an excitation source at 532 nm, provided by a frequency doubled Nd:YAG laser. The Raman probe is focused on the sample with a 60 *X* dry objective (N.A. = 0.8), providing an almost diffraction limited spot on the sample. The back-scattered radiation was collected with the same objective lens and, after spectral filtering of the Rayleigh spectral contribution by using an edge filter, it was guided toward the spectrograph by a 50 *μ*m core optical fibre, which assures the system confocality. For this system, we estimated a spatial resolution in the x-y plane (orthogonal to the Raman beam) of ∼ 300 nm, while in the z direction (microscope axis) of ∼ 800 nm. The sample is located on a piezo-electrically driven microscope scan stage with X-Y resolution of ∼ 3 nm. Spectra were acquired over the spectral range from 300-3800 cm^−1^ (1024 points), with a spectral resolution of 3.6 cm^−1^.

### Principal components analysis

The differences in spectral features of individual Raman spectra collected from biosystems owning to different groups are usually quite small and below the noise superimposed on the Raman spectra. Thus, simple spectral inspections usually reveal only a fraction of the information contained within the spectral data. In such cases, the use of multivariate methods is mandatory for extracting the small but relevant variations in the collected spectra. Among these, Principal Component Analysis (PCA) plays a relevant role [28]. The basic idea underlying PCA concept is that data variance is the best way to discriminate between data and to get an insight into the underlying phenomena observed. For this purpose, PCA decomposes the original data set into “principal components” (PCs), so that the relevant information originally contained in a large amount of variables is condensed in a very small number of PCs. PCs are defined as a linear combinations of the original data. The first PC contains the maximum variance from the data, the second PC the next highest amount, and so forth. When applied to Raman spectra, PCA condenses the information contained in each spectrum (which has a number of observables equal to its points) in only a few variables (PCs scores). The order of the PCs denotes their importance in highlighting differences within the spectra dataset, with PC1 describing the highest amount of variation. Typically, the first 3 PCs retain more than 90% of the original information, so that spectra can be represented as points in a 3-dimensional space (scores plot). In this space, spectra which are similar occupy the same region, while spectra with different features are points in quite distant regions. The bases of these groupings are revealed through the loadings, the numbers that give the contribution of each wavenumber to a particular PC. In this work, PCA was performed on Raman spectra using a home-made MATLAB routine, based on the use of the *princomp* routine. In order to correctly extract information from the acquired data, spectra were properly pre-treated. In particular, Raman spectra were background-corrected by removing a forth-order polynomial curve by using an home-made automated routine and eliminating spurious signals deriving from cosmic rays contributions.

### In vitro fertilization and culture

The in vitro fertilization medium was Tyrode’s modified medium [29] without glucose and bovine serum albumin (BSA), supplemented with 5.3 SI/mL heparin, 30 *μ*M penicillamine, 15 *μ*M hypotaurine, 1 *μ*M epinephrine and 1% of BS. Oocytes were washed and transferred, about 20-25 per well, into 300 *μ*L of IVF medium, covered with mineral oil in four well plates (NuncTM, Roskilde, Denmark). Frozen-thawed sperm of a bull previously tested for IVF were separated on Percoll discontinuous gradient (45% and 80%). The pellet was reconstituted into 2 mL of IVF medium and centrifuged twice, at 160 and 108 g for 10 min. The pellet was diluted with IVF medium and added in the fertilization wells at the concentration of 1 x 10^6^ sperm/mL. Gametes were coincubated for 20 h at 39°C, in 5% CO_2_ in air, after which presumptive zygotes were vortexed for 2 min to remove cumulus cells in H199 supplemented with 5% of BS, washed twice in the same medium, and cultured (20-25 per well) in 400 *μ*L of SOF medium [30] modified with the addition of 30 *μ*l/mL essential amino acids, 10 *μ*l/mL non-essential amino acids, 0.34 mM sodium citrate, 2.77 mM myo-inositol and 5% BS. Culture was carried out in a humidified mixture of 5% CO_2_, 7% O_2_, and 88% N_2_ in air at a temperature of 39°C. The percentage of oocyte surviving was assessed at the time of IVF (Day 0), whereas cleavage and blastocyst rates were respectively recorded on Days 2 and 7.

### Reactive oxygen species (ROS) measurements

Reactive oxygen species levels in oocytes were quantified by measuring 2’,7’-dichlorodihydrofluorescein diacetate (DCHFDA) fluorescence. A stock solution of DCHFDA was prepared in DMSO at 5 mM and stored in the dark at -20°C. Immediately before use, the stock solution was diluted to 5 *μ*M in Phosphate Buffered Saline (PBS). Samples of 48 denuded oocytes per each group (CTR, CP and VITRI), over 3 replicates, were incubated in 50 *μ*L of DCHFDA at 37°C for 30 min into a black 96-well plate (PerkinElmer Inc., Covina, CA, USA). The ROS amount was quantified by measuring the oocytes-emitted fluorescence using the fluorescein channel (1.0 s) by the Fluorescence Multilabel Reader (PerkinElmer Inc., Covina, CA, USA) at 485 nm excitation and at 535 nm emission. Correction for autofluorescence were made by including parallel blanks in each experiment. Data were expressed as arbitrary units of fluorescence (means ± SEM) relative to the CTR.

### Statistical analysis

Differences in survival, cleavage and blastocyst rates among CTR, CP and VITRI groups were analysed by ANOVA, with LSD test used for post-hoc comparison. The same analysis was used to compare ROS levels among groups.

## Results

### Raman analysis of single oocytes

**Fig 1:**
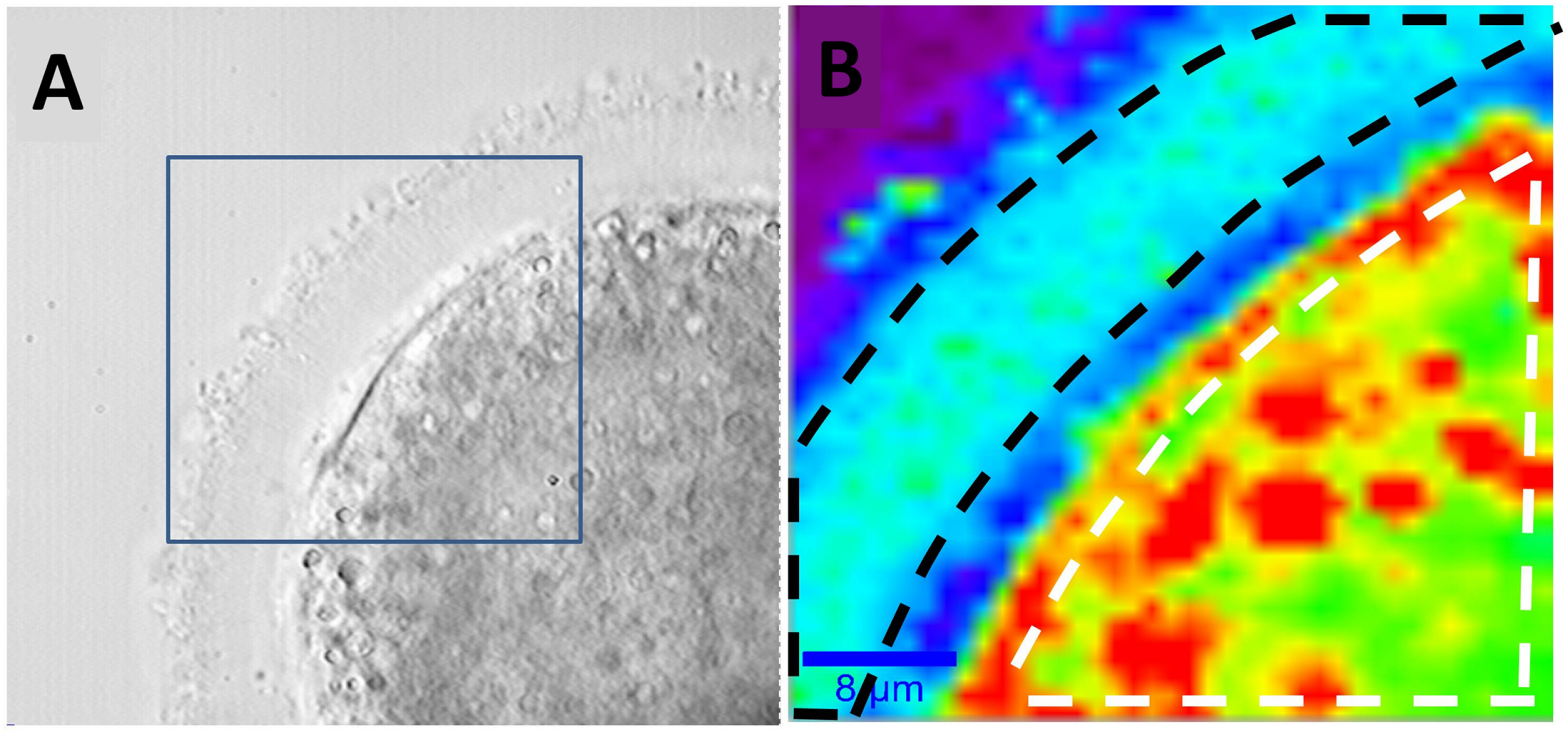
A) Bright field image of an oocyte from the control group. The blue square highlights the region analysed by Raman analysis. B) Raman image obtained *via* Raman imaging. The white dashed line defines the oocyte area used for the calculation of the average spectrum of the cytoplasm region, while the black dashed line borders the region used for the ZP region.

Oocytes from the three previously mentioned groups were analysed by Raman microscopy. At this purpose, cells were washed three times in PBS to remove the excess of paraformaldehyde, mounted on an uncoated glass coverslip and finally covered with a further glass slide. In order to reduce water evaporation, the sample cell was sealed with low-pressure vacuum grease. Typically, oocytes were analysed within a week from sample preparation. Raman imaging was performed by defining a 40 *μ*m x 40 *μ*m area including both the ZP and the cytoplasmic region. Spectra were therefore collected on a 41 x 41 points grid (step size = 1 *μ*m) with an integration time of 1s, so that a single Raman imaging required ∼ 28 min. The laser power impinging on the sample was limited to 5 mWatt to avoid photoinduced-effects. Fig 1A reports the Raman image of an oocyte from the control group acquired at approximately the cell equatorial plane. In particular, the false-colour maps report the intensity of the peak at 2880 cm^−1^ (CH symmetric stretching), mainly due to lipids. This image reproduces quite well the architectural features of cells, revealing some details not readily identifiable in the cell wide-field image (Fig 1A). In particular, the Raman image clearly highlights the presence of the oolemma and some lipid grains (coloured in red), which correspond to points with the higher intensity of the CH stretching band.

**Fig 2:**
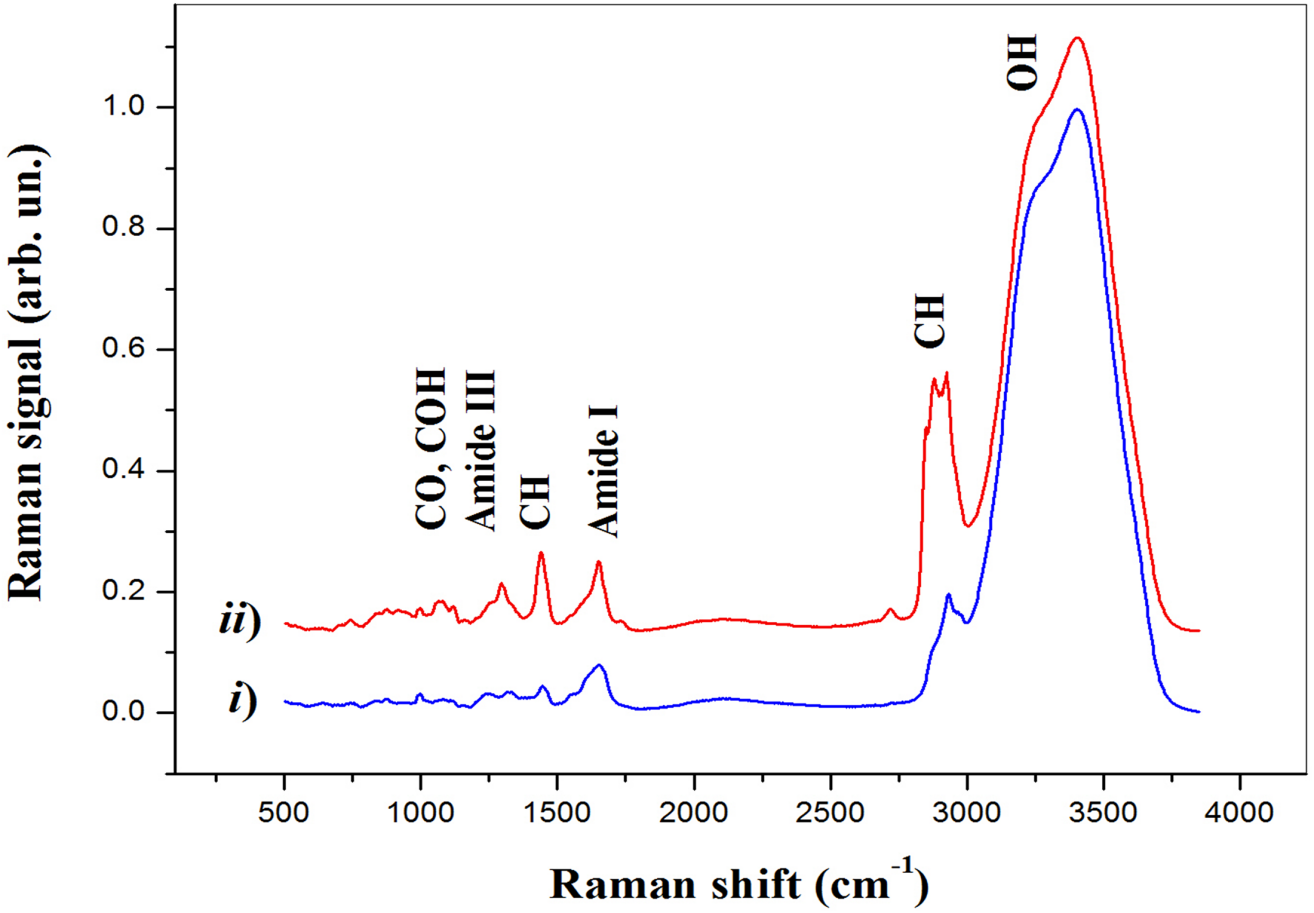
Average spectra from the ZP (*i*) and cytoplasm (*ii*) obtained for the oocyte shown in Fig 1.

At the same time, the different lipid content in the oocyte compartments allows the differentiation of the ZP (coloured in blue), corresponding to areas with the less pronounced lipid content and the cytoplasmic matrix (colored in green). Similar results were obtained for oocytes belonging to all the classes investigated in this work. In order to investigate the biochemical differences among oocytes of different groups, for each oocyte the mean spectra from the cytoplasm and ZP was extracted. This operation was easily carried out with the help of the WiTech Project software, allowing to perform the average spectrum of the Raman signals acquired in a user-defined area. For example, in Fig 1B the white dashed line defines the oocyte area used for the calculation of the average spectrum of the cytoplasm region, while the black dashed line borders the region used for the ZP region. Both average spectra are reported in Fig 2. They were normalised to the height of the prominent features at ∼ 3400 cm^−1^ due to water. Being these traces the mean spectra of hundreds of spectra, the signal quality is quite good, and one can immediately discern the spectral differences between the average from the ZP (which exhibits mostly protein features) and that from the cytoplasm region (in which the protein features are mixed with quite pronounced lipid-related features). Table 2 reports the assignment of the main spectral features observed in our analysis. Notably, the spectral regions around 1250 cm^−1^ and 1650 cm^−1^ show the protein conformation sensitive bands Amide I and Amide III, respectively. Instead, information regarding the lipids can be mainly obtained by the CH bending (around 1440 cm^−1^) and CH stretching (around 2880 cm^−1^) bands, as well as the C = C band at 1655 cm^−1^ characteristic of unsaturated lipids. Finally, the CO and COH bands in the 1020-1210 cm^−1^ region provides information on the carbohydrates presence in the different oocyte compartments.

**Table 2:** Assignment of the prominent Raman features observed in our investigation.

Wavenumber (cm^−1^)	Assignment
748	Trp
930	C-C stretch
830, 850	Tyr
1001, 1030	Phe
1091	C-C stretch
1137	C-O-H stretch
1205	C-O-C stretch
1220-1270	Amide III
1363	Trp
1440	*CH* bend
1510-1530	Amide II
1580	Phe, Trp
1620-1650	Amide I
1655	*C* = *C* stretch
1735	*C* = *O* stretch
2880	*CH* stretch
3400	*OH* stretch

### Differentiation among control, cryoprotected and vitrified oocytes

As previously stated, the main focus of this work was the investigation of the structural modifications of ZP and cytoplasm of vitrified-warmed bovine oocytes, together with those associated to the simple exposure to CP. However, the spectroscopic effect of this transformation was not immediately detectable, being masked by the inherent heterogeneity associated with the analysed bio-system. In particular, the average signals for both ZP and cytoplasm showed no significant difference among the groups considered in this investigation. Therefore, we choose to analyse our set of data by PCA. For this purpose, the average spectra obtained from oocytes were normalised, mean centred and finally decomposed in PCs. As described below, this approach allowed us to unravel significant difference among groups, which were associated to the biochemical transformations induced by vitrification and/or the use of CPs.

#### Zona pellucida

**Fig 3:**
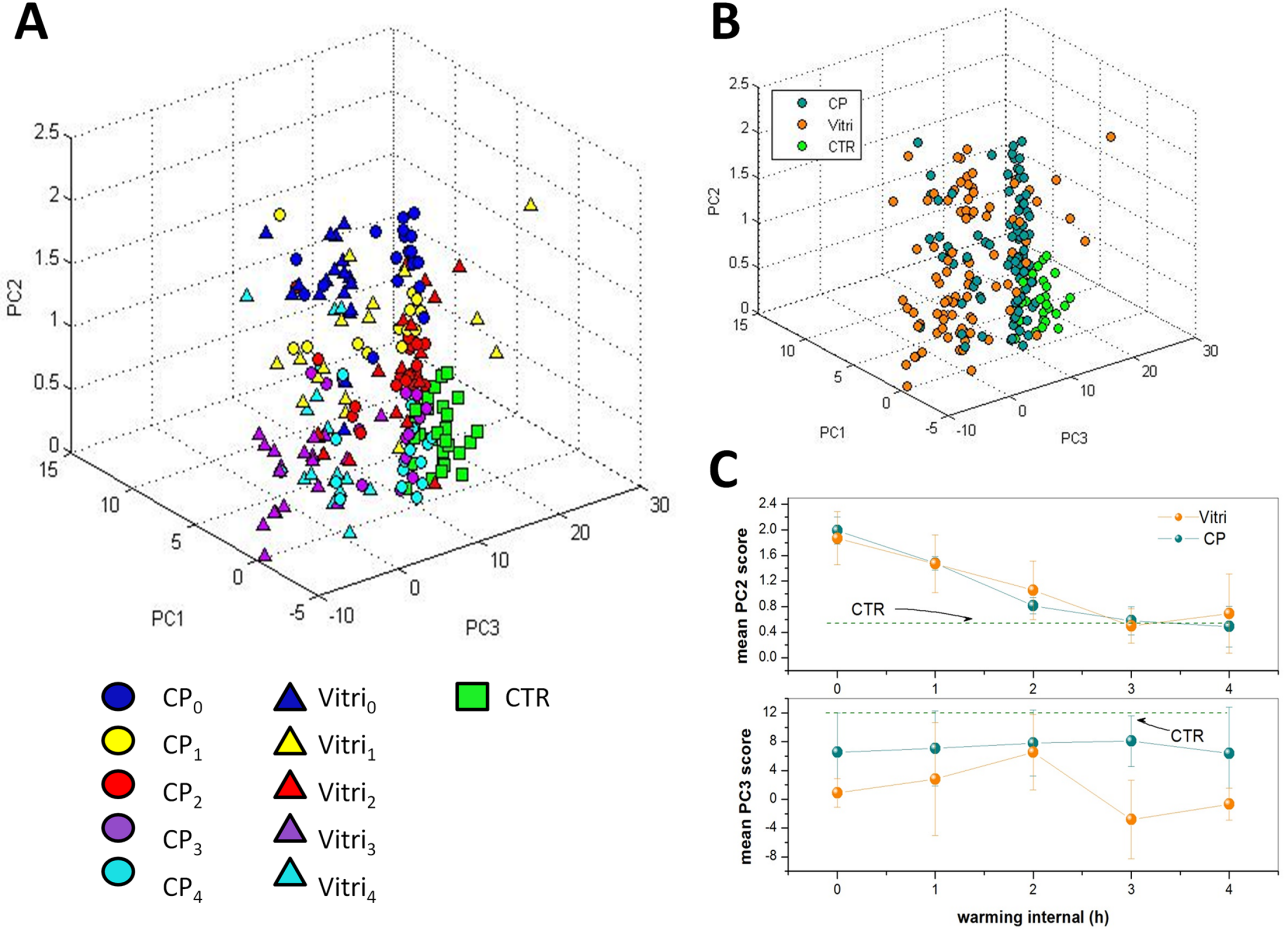
A) Score plot relative to the PC analysis of spectra from the ZP of cells specified in Table 1. The symbols corresponding to CP_*i*_ and VITRI_*i*_ are dots and triangles, respectively, while spectra corresponding to the CTR group are represented by squares. For both CP and VITRI groups, the different colours indicate the different warming intervals before cells fixation. B) Same score plot as in part a) but with points corresponding to the same CP_*i*_ and VITRI_*i*_ group (i = 1-4) represented by the same symbol. C) Mean PC2 and PC3 scores for all the groups analyzed herein. The bars indicated represent the standard deviations obtained for each group.

**Fig 4:**
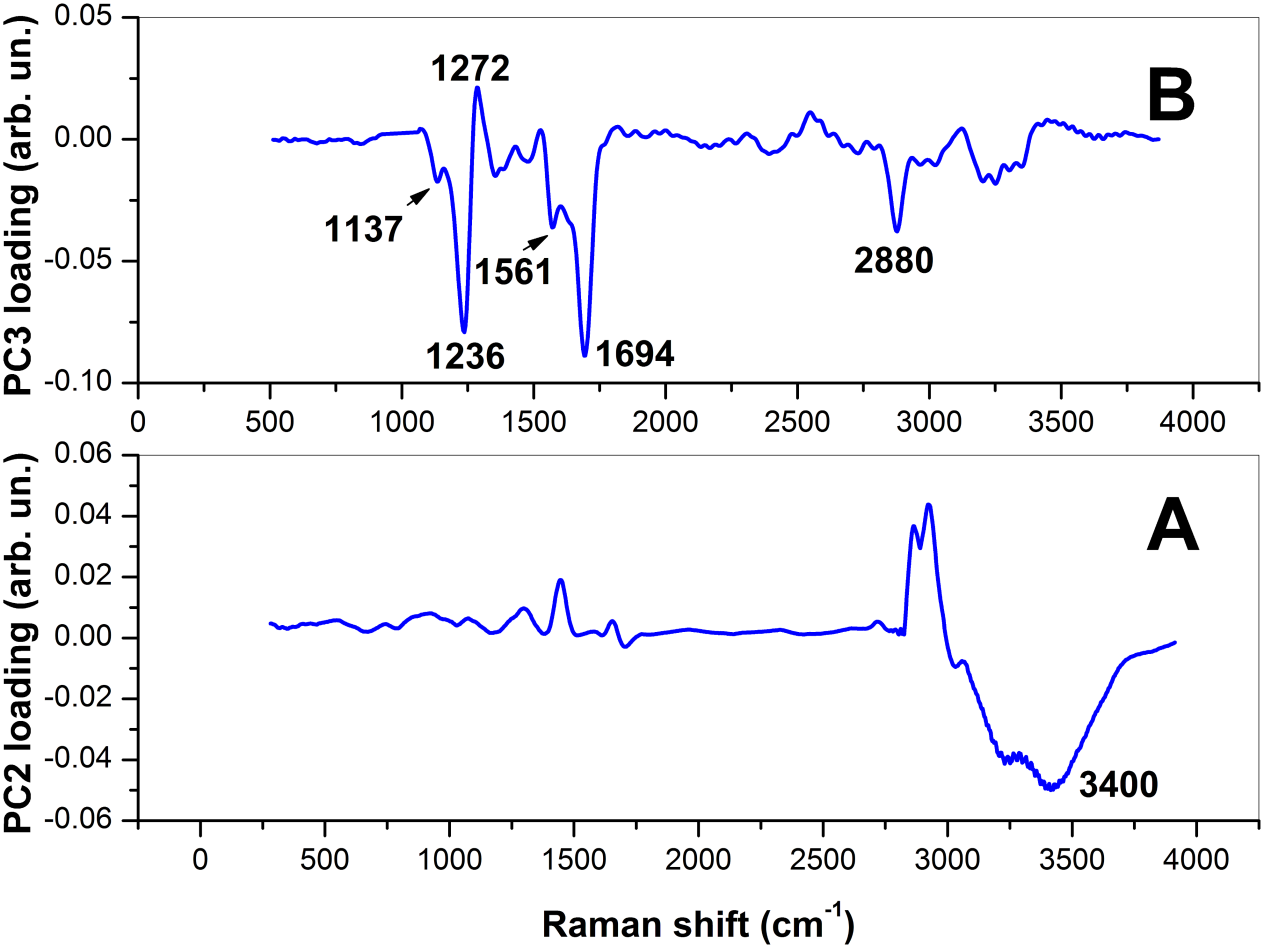
PC2- (A) and PC3-loading (B) plot resulting from the analysis of all the 195 average spectra from the ZP.

Fig 3A shows the three-dimensional score plot resulting from the analysis of the spectra for the first 3 PCs, which globally take into account ∼ 85% of the total spectra variability. The symbols corresponding to CP_*i*_ and VITRI_*i*_ are dots and triangles, respectively, while spectra corresponding to the control group are represented by squares. For both CP and VITRI groups, the different colors correspond to the different warming intervals before cells fixation. Interestingly, while PC1 does not exhibit any differentiation among the groups, there is a clear tendency of points owning to the same group to cluster along the PC2 coordinate and, to a lesser extent, to the PC3 coordinate. Notably, PCA provides an efficient clusterization of points from the control group, for which can be intuitively expected a relatively lower variability. Most of differentiation among groups has been achieved along the PC2 coordinate. To better highlight this effect, in Fig 3C we report the mean PC2 score (*μ*_*i*_), along with the standard deviation, for data from the same group. Intriguingly, for both CP and VITRI groups, there is a clear decreasing of *μ*_*i*_ with the warming interval, up to t = 2 h where a plateau level is reached. The reason of this trend can be easily understood by PC2 loading inspection (Fig 4). This loading exhibits some unspecific positive features in the 500-2000 cm^−1^ and 2700-3200 cm^−1^, and a negative feature around 3400 cm^−1^, associated to the water Raman bands. Therefore, it is reasonable to think that PC2 loading takes into account the oocyte dehydration occurring as an effect of CPs exposure prior vitrification and subsequent rehydration occurring during the warming process. In this frame, due to the negative sign of the water band, the lower PC2 score values correspond to the higher presence of water; according to this outcome the water content seems to be completely re-established in VITRI and CP groups within 2 h post-warming. Coherently, CTR oocytes, which were not exposed to CPs, exhibit the lower PC2 scores values. As PC2, also PC3 component provides some differentiation among the groups. In particular, the score plot highlights a clear differentiation between CTR and CP oocytes on one side, and VITRI cells on the other side. This effect is highlighted in Fig 3B, where points corresponding to all the VITRI_*i*_ and CP_*i*_ (i = 1-4) are represented by the same symbol. Therefore, contrary to PC2, PC3 component seems to take into account some biochemical effect related to the vitrification process more that to the use of cryoprotective agents. As first outcome, it is worth noticing that while points corresponding to CTR and, to a less extent, also CP and VITRI_0_, are well confined along the PC3 coordinate, the other groups exhibit a wider PC3 score dispersion. In particular, VITRI2 points occupy a wider region, with most of them overlapped to the same high PC3 score values filled also with CTR points (see Fig 3B). The PC3 loading is quite complex, presenting both positive and negative features. The positive loading feature lies around 1272 cm^−1^ and can be attributed to proteins assembled in an *α*-helices configuration. The negative features can be attributed to proteins arranged as *β*-sheets (features around 1236 cm^−1^ and 1561 cm^−1^), carbohydrates (1137 cm^−1^ and 1694 cm^−1^), as well as lipids (around 2880 cm^−1^). In particular, this latter peak characterizes lipids exhibiting well-packed acyl chains configuration [31]. Clearly, the counter-variation of the protein secondary structure highlighted by PC3 loading with points corresponding to vitrified oocytes exhibiting the lower PC scores, suggests that, upon the vitrification-warming process, ZP acquires the more-ordered *β*-sheets structure, which contributes to its well-known hardening. However, the presence of the lipid-related feature around 2880 cm^−1^ suggests that the vitrification-warming procedure also induces some packing effect on lipids, which reasonably contribute to the global ZP mechanical hardening.

#### Cytoplasm

**Fig 5:**
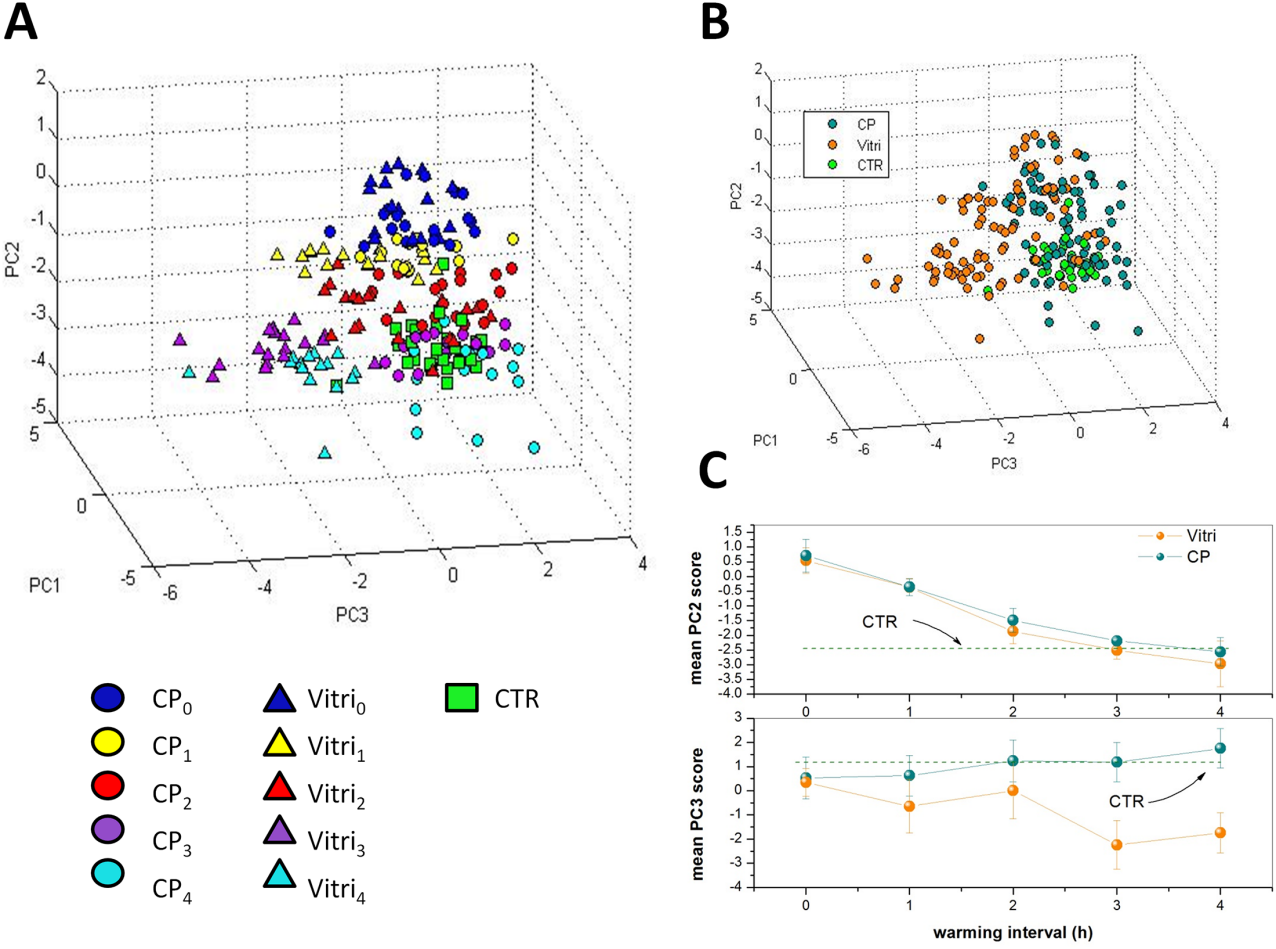
A) Score plot relative to the PC analysis of the oocytes cytoplasm, as specified in Table 1.The symbols corresponding to CP_*i*_ and VITRI_*i*_ are dots and triangles, respectively, while spectra corresponding to the control group are represented by squares. For both CP and VITRI groups, the different colours indicate the different warming intervals before cells fixation. B) Same score plot as in part a) but with points corresponding to the same CP_*i*_ and VITRI_*i*_ group (i = 1-4) represented by the same symbol. C) Mean PC2 and PC3 scores for all the groups analyzed herein. The bars indicated represent the standard deviations obtained for each group.

**Fig 6:**
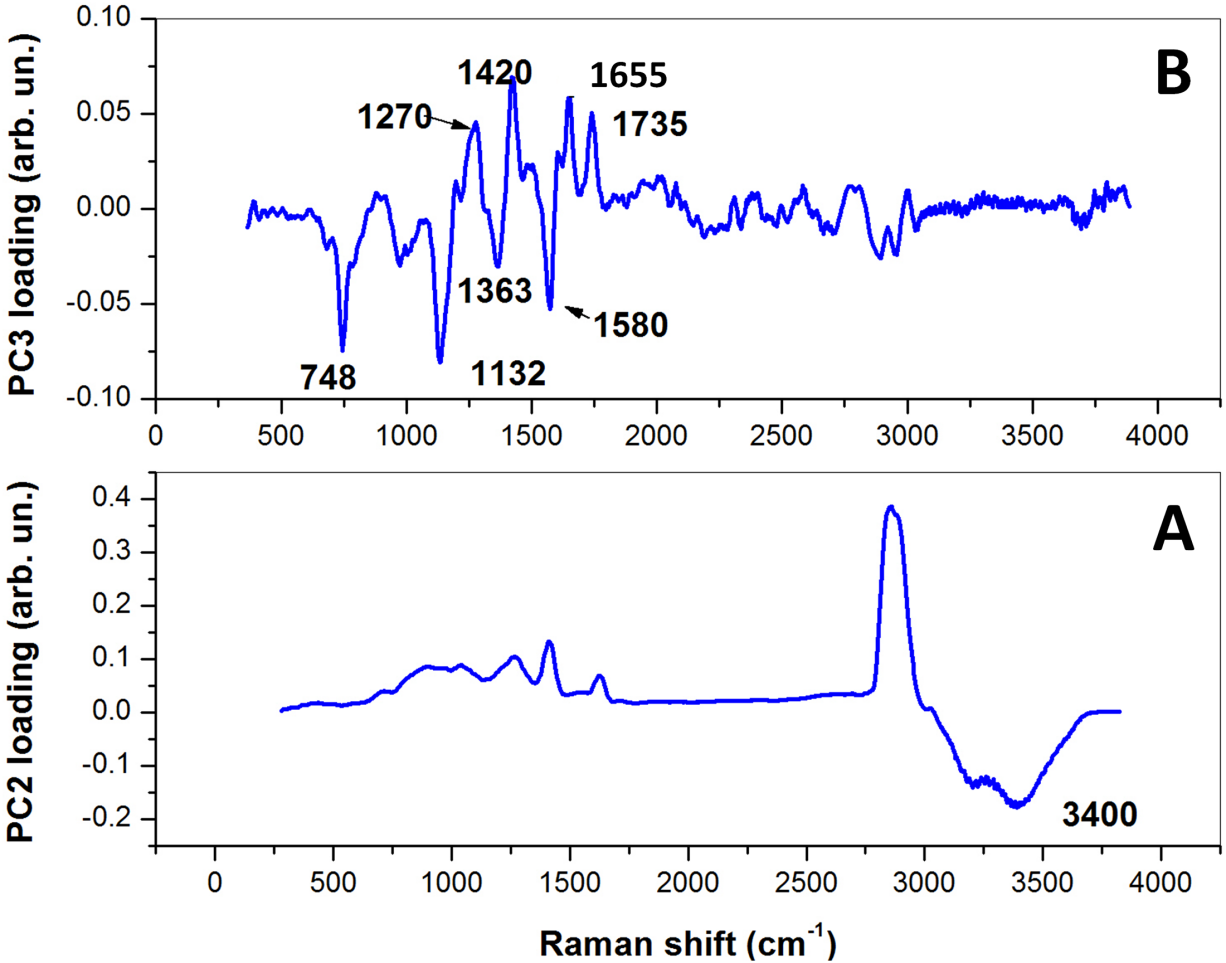
PC2- (A) and PC3-loading (B) plot resulting from the analysis of all the 195 average spectra from the cytoplasm.

Fig 5 reports the score plot obtained for the first three PC components obtained by the analysis of the samples specified in Table 1. The symbols-groups association is the same reported in Fig 3. The differentiation among groups observed for the ZP is clearly reduced in the case of cytoplasm. The only reasonable differentiation is along the PC2 score coordinate which, on the light of the features exhibited by the respective loading (Fig 6), seems to take into account the water recovery by the cells during the warming process observed for the ZP. However, while PC1 does not provide any differentiation among groups, PC3 suggests biochemical differences between CTR and CP on one side and VITRI groups on the other side, particularly at 3 h and 4 h post-warming. PC3 loading exhibits some negative, sharp peaks assignable to essential amino acids, and positive features assignable to *α*-helices proteins (around 1270 cm^−1^) and lipids (around 1420 cm^−1^, 1650 cm^−1^ and 1735 cm^−1^). Therefore, the presence of the positive peak at 1270 cm^−1^ suggests a reduced *α*-helices content of vitrified oocytes. Notably, according to the PC3 loading, VITRI cells can be distinguished from CTR and CP cells also by their lipid content. In particular, the presence of the band at 1650 cm^−1^ due to the C = C bond reveals a lower concentration of unsaturated lipids in VITRI oocytes [32].

#### In vitro fertilization and culture

Despite similar survival rates, both cleavage and blastocyst rates decreased (P<0.01) in CP and VITRI groups compared to the CTR group (Table 3). In addition, VITRI oocytes gave lower cleavage (P<0.01) and blastocyst (P<0.05) rates than CP oocytes (Table 3).

**Table 3:** Survival rates at the time of IVF, cleavage and blastocyst rates from oocytes of CTR, CP and VITRI groups.

Groups	n.	Survival	Cleavage	Blastocyst rate
		Mean ± SE	± SE	± SE
Control	183	100.0 ± 0.1	83.8 ± 0.7^A^	52.2±1.4^A^
CP	218	94.4 ± 2.7	67.6 ± 1.2^B^	25.2±4.1^B^^,^^a^
VITRI	218	92.2 ± 6.9	33.9 ± 2.0^C^	10.1±1.7^B^^,^^b^

^*A*,*B*,*C*^ Values with different superscripts are significantly different; P< 0.01

^*a*,*b*^ Values with different superscripts are significantly different; P<0.05

#### ROS measurements

**Fig 7:**
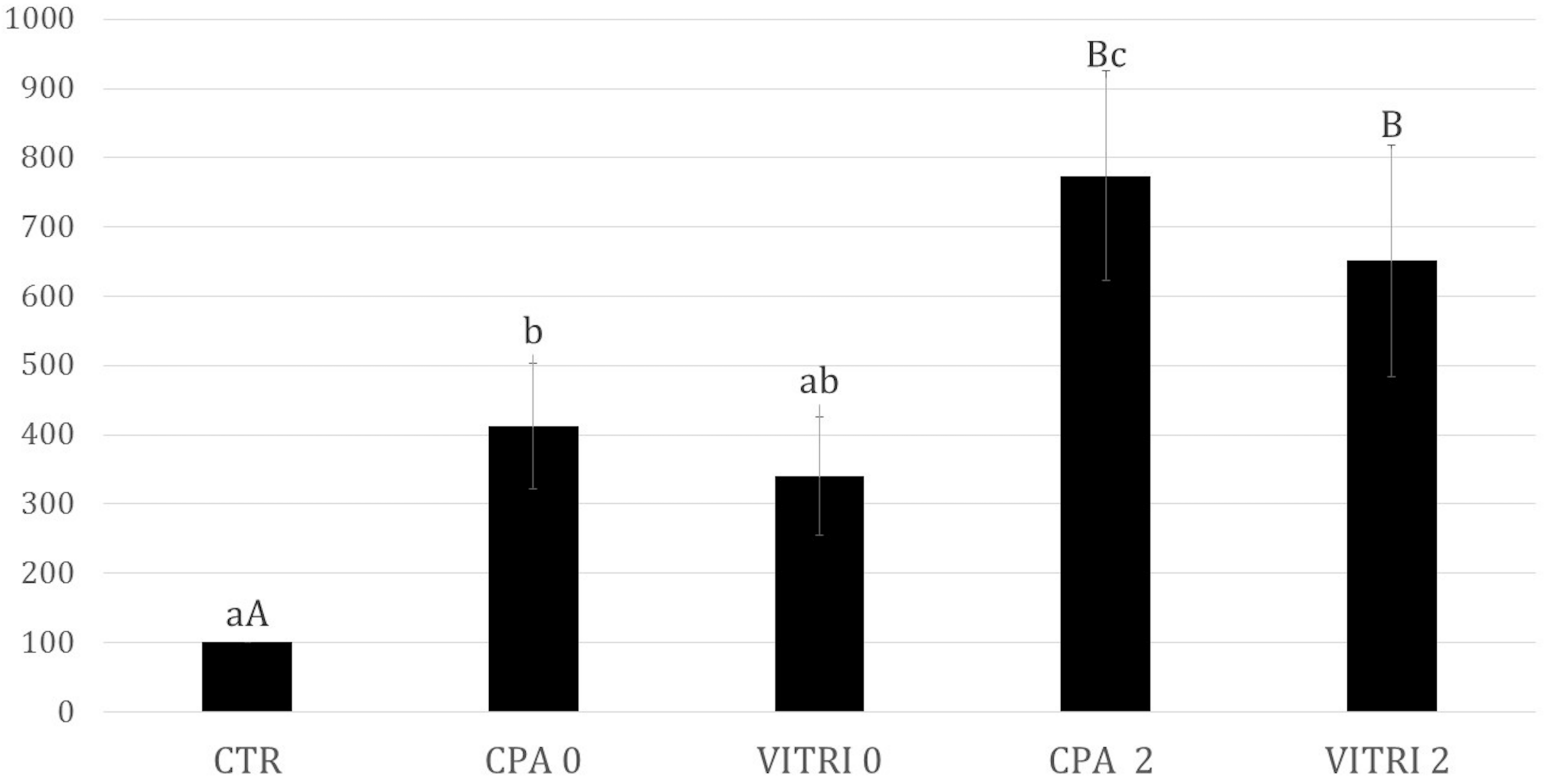
ROS levels (Units of fluorescence) in matured bovine oocytes vitrified (VITRI) and exposed to cryoprotectants (CP) at 0 and at 2 h post-warming compared to the control (CTR). ^*A*,*B*^ Values with different superscripts are significantly different; P< 0.01. ^*a*,*b*,*c*^ Values with different superscripts are significantly different; P<0.05.

The simple exposure of oocytes to the CPs increased (P<0.05) the ROS levels compared to the CTR, whereas intermediate values were recorded in VITRI oocytes at time 0 Fig 7. However, at 2 h post-warming both VITRI and CP oocytes displayed higher (P<0.01) ROS levels than CTR oocytes. No differences were observed between VITRI and CP groups within the same time. In addition, oocytes of the CP group at 2 h post-warming had higher (P<0.05) ROS values than both CP and VITRI oocytes at time 0.

## Discussion

In the present study, the biochemical modifications of both ZP and cytoplasm of vitrified/warmed in vitro matured bovine oocytes at different post-warming times were assessed by RMS. The spectroscopic investigation shown herein provides several interesting outcomes on the rearrangement of oocyte biochemical architecture following vitrification-warming, and suggests that the biochemical modifications are in part reversible after 2 h post-warming. The most relevant modifications were observed in vitrified-warmed oocytes (VITRI group) compared to the control, with oocytes only exposed to vitrification and warming solutions (CP group) exhibiting more similar patterns to control untreated oocytes (CTR group). The first difference observed between CTR oocytes and both CP and VITRI oocytes is related to water content both at the level of ZP and cytoplasm. Indeed, the water content of CP and VITRI oocytes at time 0 is lower than in CTR oocytes, to increase at subsequent times, reaching a plateau level after 2 h. This is likely due to the oocyte dehydration occurring as an effect of CPs exposure prior vitrification and subsequent rehydration occurring during the warming process. These results suggest that the recovery of the physiological water level in both ZP and cytoplasm requires 2-3 h from the starting of the warming phase. Interestingly, in human oocytes the completion of rehydration occurs after 3 h culture [33]. This is in line with the usual protocols of laboratory for oocytes fertilization, which typically includes about a 2 h warming phase before oocytes exposure to spermatozoa [34, 35].

However, other relevant modifications were observed at both ZP and cytoplasmic levels.

With regard to ZP, a rearrangement of the protein secondary structure occurred in vitrified oocytes, indicated by the increase in *β*-sheet content at the expenses of the *α*-helices. Interestingly, similar conformational changes of the ZP protein secondary structure were reported in ovine oocytes following vitrification [14]. This protein transformation to a more ordered structure is in agreement with the cryopreservation-induced ZP mechanical hardening previously reported elsewhere [36, 37]. This conformational change, likely related to the premature release of cortical granules, undoubtedly contributes to ZP hardening. In fact, cryopreservation leads to premature release of cortical granules that in turn determines ZP hardening, known to impair sperm penetration [13, 37–39]. It has also been reported that the simple exposure of oocytes to CPs may result in premature release of cortical granules [39–41]. However, these structural modifications of ZP were not observed in CP oocytes, as also previously shown in ovine oocytes [14]. This suggests that the combination of EG and DMSO, in the presence of sucrose, does not affect the ZP protein secondary structure.

Nevertheless, both cleavage and blastocyst rates after IVF decreased also in CP oocytes, although to a less extent. This may be accounted for by other alterations induced by the toxicity of CPs, via osmotic stress, such as extreme fluctuations in cell volume, membrane and cytoskeleton damage, abnormal chromatin and meiotic spindle configuration [42–44]. Furthermore, lipid configuration of ZP also changed in relation to the treatment. Although the ZP was previously thought to be mainly composed by glycoproteins [45, 46], it has became evident that this extracellular coat is also composed of different lipids [47–49]. Our results suggest that vitrification results in a more packed structure of ZP lipids [31] in vitrified oocytes, contributing to the ZP hardening.

Finally, modifications of carbohydrate components of ZP glycoproteins were observed in vitrified oocytes, as suggested by the differences in the bands that peaked at 1137 cm^−1^ and 1694 cm^−1^. Modifications of carbohydrates were also previously reported in ovine oocytes following vitrification [14]; in particular a difference between VITRI and CTR was found in the band that peaked at around 1137 cm^−1^, attributed to sialic acid [50] in both studies. With regard to the cytoplasm, the most relevant modifications were recorded between CTR and CP from one side and VITRI oocytes. Vitrified oocytes underwent a modification of the secondary structure of cytoplasmic proteins, with a decreased *α*-helices content. This may be the result of cold-induced protein denaturation. In addition, our results highlighted that also the unsaturated lipid content is lower in VITRI oocytes with respect to both CTR and CP oocytes. The decrease of C = C bonds may be due to the cryoinjury itself [51], as well as to reactive oxygen species (ROS) attack [52]. It is known that increased oxidative stress occurs during cryopreservation [53–55] and after exposure to CPs [56]. Accordingly, in the present study ROS levels increased both in CP and VITRI oocytes. However, as ROS production was similar in CP and VITRI oocytes, the altered spectrum associated to decreased lipid unsaturation is likely due to other vitrification-associated events rather than oxidative stress. Our outcomes also suggest, at cytoplasmic level, a higher essential amino acids concentration in VITRI oocytes compared to CTR and CP oocytes. This finding is difficult to explain. It is not possible to rule out that, due to the release of cortical granules, an event observed in oocytes following the vitrification process, a modification of the cytosol proteins composition occurs. Clearly, an insight on this hypothesis could be obtained only on the basis of the precise knowledge of proteins contained in the released cortical granules, which is out of the scope of the present work. All the biochemical modifications highlighted by RMS both at the ZP and cytoplasm compartments in vitrified oocytes may account for the marked decrease of the oocyte developmental competence, indicated by a severe reduction of cleavage and blastocyst rates. It is worth noting that also the simple exposure to CPs partially results in a decreased oocyte competence, that is though not associated to altered RMS spectra. Furthermore, our experimental outcomes confirm that the optimal post-warming time for in vitro fertilization of vitrified bovine oocytes is around 2 h, indicated by both the accomplishment of rehydration process and the more evident biochemical modification observed at subsequent times. The obtained results confirm the effectiveness of RMS for quality assessment of cells following the vitrification process. However, further studies are required for the analysis of viable and vulnerable samples, such as live human oocytes, that can be reasonably accomplished by a significant reduction in the time required for spectral acquisition on the sample.

## Conclusion

In conclusion, Raman spectroscopy combined with PCA was used to assess the biochemical modifications that occur following vitrification of in vitro matured bovine oocytes both at the zona pellucida and cytoplasm levels. It was demonstrated that vitrification alters the protein secondary structure, with an increase of the *β*-sheet content at the expenses of the *α*-helices, as well as the lipid and carbohydrate configuration of the ZP. These changes are compatible with cryopreservation-induced zona hardening, known to impair fertilization. It was also demonstrated that at the cytoplasmic levels modifications occur in protein secondary structure, with *α*-helices loss, suggesting cold protein denaturation, and a decrease of C = C bonds, likely associated to cryoinjury. Furthermore, most modifications were not observed in oocytes exposed to CPs, suggesting that the combination of CPs does not severely affect the biochemical architecture of the oocyte.
